# Emergence of influenza B/Victoria in the Micronesian US-affiliated Pacific Islands, spring 2019

**DOI:** 10.5365/wpsar.2021.12.4.706

**Published:** 2021-10-27

**Authors:** Stephanie O’Connor, W. Thane Hancock, Estelle Ada, Edlen Anzures, Christine Baza, Annette L. Aguon, Doris Cruz, Eliaser Johnson, Allan J. Mallari, Jill A. McCready, Jack Niedenthal, Ann Pobutsky, Anne Marie Santos, Jose Villagomez Santos, Jeremy Sasamoto, Portia Tomokane, Warren Villagomez, Paul White

**Affiliations:** a Hubert Department of Global Health, Rollins School of Public Health, Emory University, Atlanta, GA, United States of America.; b Career Epidemiology Field Officer Program, Division of State and Local Readiness, Center for Preparedness and Response, United States Centers for Disease Control and Prevention, Atlanta, GA, United States of America.; c Department of Public Health and Social Services, Mangilao, Guam.; d Ministry of Health and Human Services, Majuro, Republic of the Marshall Islands.; e Public Health and Hospital Emergency Preparedness Program, Commonwealth Healthcare Corporation, Saipan, Commonwealth of the Northern Mariana Islands.; f Department of Health and Social Affairs, Pohnpei, Federated States of Micronesia.; g Immunization Program, Commonwealth Healthcare Corporation, Saipan, Commonwealth of the Northern Mariana Islands.

## Abstract

Data collected through routine syndromic surveillance for influenza-like illness in the Micronesian United States-affiliated Pacific Islands highlighted out-of-season influenza outbreaks in the spring of 2019. This report describes the data collected through the World Health Organization’s Pacific Syndromic Surveillance System for the Commonwealth of the Northern Mariana Islands (CNMI), Guam, the Federated States of Micronesia (FSM) and the Republic of the Marshall Islands (RMI). Compared with historical data, more cases of influenza-like illness were observed in all four islands described here, with the highest number reported in Guam in week 9, CNMI and FSM in week 15, and RMI in week 19. The outbreaks predominantly affected those aged < 20 years, with evidence from CNMI and RMI suggesting higher attack rates among those who were unvaccinated. Cases confirmed by laboratory testing suggested that influenza B was predominant, with 83% (99/120) of subtyped specimens classified as influenza B/Victoria during January–May 2019. These outbreaks occurred after the usual influenza season and were consistent with transmission patterns in Eastern Asia rather than those in Oceania or the United States of America, the areas typically associated with the United States-affiliated Pacific Islands due to their geographical proximity to Oceania and political affiliation with the United States of America. A plausible epidemiological route of introduction may be the high levels of international tourism from Eastern Asian countries recorded during these periods of increased influenza B/Victoria circulation. This report demonstrates the value of year-round surveillance for communicable diseases and underscores the importance of seasonal influenza vaccination, particularly among younger age groups.

The United States-affiliated Pacific Islands are a group of six countries and territories spread across the Pacific. In spring 2019, unusual increases in influenza-like illness (ILI) were reported in four of these Micronesian islands: the Commonwealth of the Northern Mariana Islands (CNMI); the Federated States of Micronesia (FSM), comprising the states of Chuuk, Kosrae, Pohnpei and Yap; Guam; and the Republic of the Marshall Islands (RMI) (**Fig. 1**).

These islands are part of the World Health Organization’s (WHO’s) Pacific Syndromic Surveillance System, which monitors ILIs and other syndromes and distributes weekly reports with data from 23 participating Pacific island countries and territories. (*1*) Despite inclusion in surveillance system dispatches, these 23 countries and territories have low representation in broader regional reports, partly because of their limited diagnostic testing capacity as well as their small populations, which are dwarfed by other members of WHO’s Western Pacific Region. Data from these US-affiliated islands also generally do not appear in United States influenza surveillance reports. As a result, surveillance of the burden, distribution and type of influenza impacting the Pacific island countries and territories may be incomplete. This report uses surveillance data from four of the Micronesian islands affiliated with the United States to expand understanding of how these islands fit into broader regional and global influenza transmission trends.

**Figure 1:**
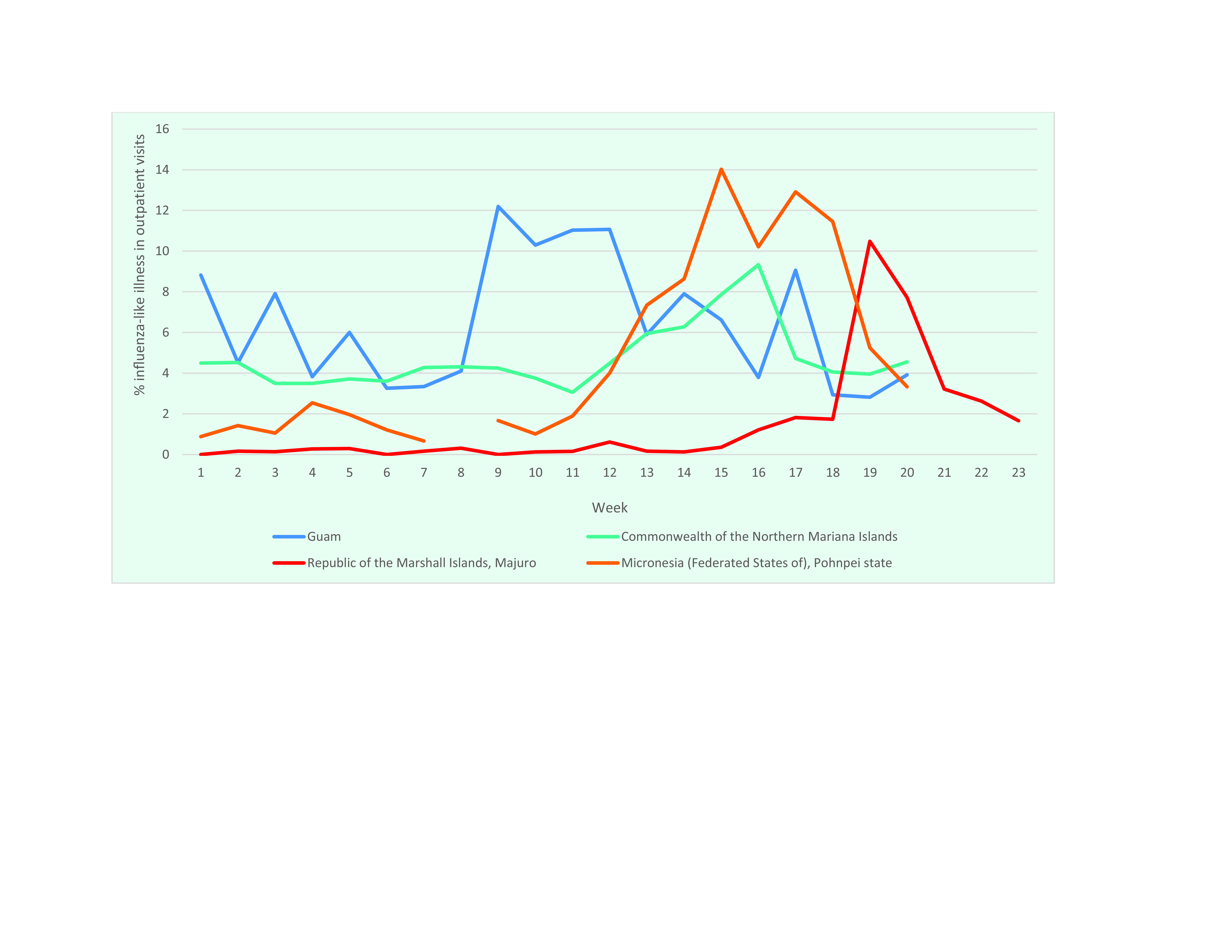
Number of cases of influenza-like illness reported in four of the US-affiliated Pacific Islands: the Commonwealth of the Northern Mariana Islands, the Federated States of Micronesia, Guam and the Republic of the Marshall Islands, weeks 1–23, 2019

[**insert** Figure 1]

## Ethics statement

This project was determined to be exempt from review by the Emory University Institutional Review Board.

## Methods

This surveillance report describes trends in ILI and influenza for weeks 1–20 of 2019 from data reported to the surveillance system from the following four US-affiliated islands: CNMI, FSM, Guam and RMI. Although the primary focus is on the time from January through mid-May, data are provided through June for RMI, which experienced a later outbreak.

ILI counts were collected as part of routine surveillance system reporting, which defines ILI as the acute onset of fever (38 °C/100.4 °F) accompanied by cough or sore throat, or both. (*1*) CNMI routinely calculates ILI rates as a percentage of total outpatient encounters; rates were calculated retrospectively for Guam, FSM and RMI.

Cases were confirmed by nasopharyngeal swab testing, which is implemented routinely on a selection of patients presenting with flu-like symptoms. Testing is done at the health-care provider’s discretion but may be more likely when providers are aware of increased circulation of influenza. A small number of nasopharyngeal swab specimens from CNMI, FSM and RMI were subtyped using reverse transcription polymerase chain reaction (RT–PCR) analyses (Applied Biosystems 7500 Fast Dx Real-Time PCR, ThermoFisher Scientific, Carlsbad, CA, USA) conducted by the Guam Public Health Laboratory and the Hawaii State Laboratories Division. The laboratory in Guam routinely selects at least four nasopharyngeal swab specimens for surveillance each week.

A confirmed influenza case was defined as infection in a patient with symptoms of ILI and a nasopharyngeal swab specimen positive for influenza by rapid or RT–PCR testing. Cases were considered probable if not confirmed through nasopharyngeal swab testing.

Data from CNMI came from seven sentinel sites on the three permanently inhabited Northern Mariana Islands. Forty-two facilities representing the four states of FSM contributed syndromic data, but ILI rates reported here are from only the eight sentinel sites in Pohnpei, which had the most complete data about total encounters. Syndromic data from Guam were collected at the island’s only public hospital, and confirmed cases were detected through electronic laboratory reports and morbidity reports from health-care facilities across the island. The RMI system is composed of hospitals and clinics located on Ebeye Island, Majuro and the Outer Islands, although the data presented here are drawn only from Majuro’s three sentinel sites due to constraints on data access. For each jurisdiction, vaccination rates were calculated based on immunization programme records, where available.

Regional trends were assessed based on information from FluNet, WHO’s online platform that aggregates influenza counts from the Global Influenza Surveillance and Response System (GISRS). (*2*)

## Results

### Guam

**Figure 2:**
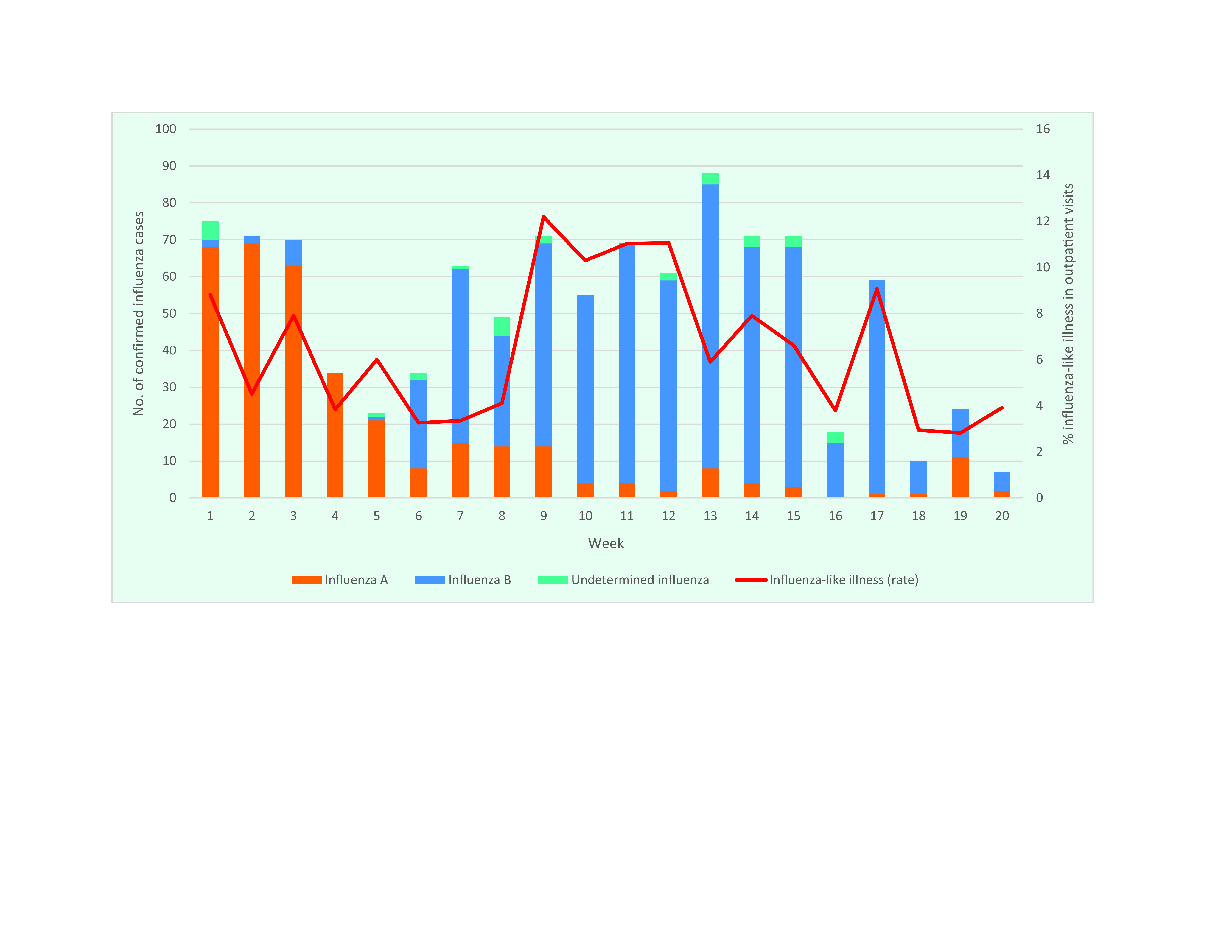
Number of cases of influenza-like illness reported and confirmed influenza, by virus type and rate, Guam, weeks 1–20, 2019

In late February and most of March 2019, Guam experienced an increase in rates of ILI (**Fig. 2**) not expected based on historical data. In weeks 8 and 9, the rate of ILI increased nearly threefold to reach 12.2% (35/287) of outpatient encounters, and it remained above 10% through week 12 (31/301, 30/272 and 29/262 in weeks 10, 11 and 12, respectively). A total of 107 specimens were randomly selected for serotyping from week 1 to 20. Although influenza A(H3N2) and A(H1N1)pdm09 were detected early in the year, the number of confirmed cases caused by influenza A generally declined beginning in late January. Cases caused by influenza A viruses reached a low just as the number of confirmed cases caused by influenza B viruses began to increase in week 6, when they represented 71% (24/34) of confirmed cases. By the peak of the outbreak in week 13, influenza B viruses accounted for 88% (77/88) of confirmed cases. Influenza B/Victoria was present in 100% of specimens tested by RT–PCR during weeks 10–20. During the full study period, 80% (86/107) of confirmed cases were caused by the Victoria lineage (**Table 1**). No influenza B/Yamagata viruses were detected.

[**insert** Figure 2]

**Table 1:** Number of positive influenza specimens by subtype, Guam, weeks 1–20, 2019^a^

Week	Influenza type		Total no. of specimens tested
	A(H1N1)pdm09 and A(H3N2)	B/Victoria	
1	8	0	8
2	–	–	–
3	–	–	–
4	–	–	–
5	4	0	4
6	5	3	8
7	0	11	11
8	1	8	9
9	3	6	9
10	0	6	6
11	0	7	7
12	0	6	6
13	0	5	5
14	0	6	6
15	0	6	6
16	0	4	4
17	0	5	5
18	0	4	4
19	0	5	5
20	0	4	4
**Total**	**21**	**86**	**107**

^a^ The Guam Public Health Laboratory subtypes a random selection of nasopharyngeal swab specimens each week for routine influenza surveillance. No testing was conducted during weeks 2–4, indicated by –.

The majority of cases of ILI occurred among those aged < 20 years. From week 6 onwards, 61% (172/280) of ILI encounters were with those aged 0–4 years, and 19% (52/280) were with those aged 5–19 years. Among confirmed influenza B cases of known-age, 70% (163/232) were 5–19 years, 20% (46/232) were < 5 years and 1 was > 50 years. Altogether, 35% (30/86) of confirmed cases classified as caused by influenza B/Victoria occurred in persons aged < 5 years, and 55% (47/86) occurred in persons aged 5–19 years.

There were six hospitalizations for confirmed cases of influenza B in weeks 5–17, with two in week 9. Five of these confirmed cases were aged £6 years, and one of these passed away after admission.

### Commonwealth of the Northern Mariana Islands

**Figure 3:**
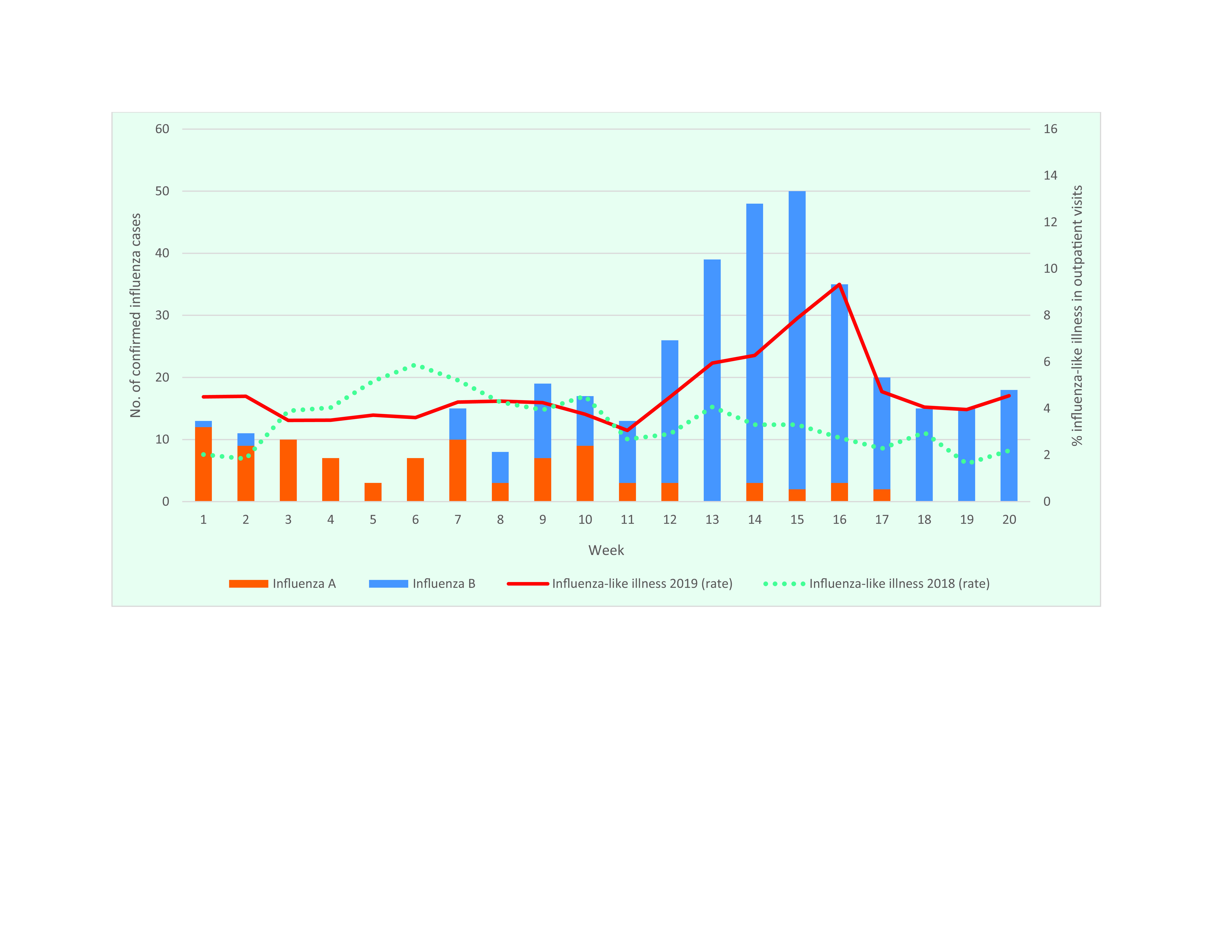
Number of cases of influenza-like illness reported and confirmed influenza, by virus type and rate, Commonwealth of the Northern Mariana Islands, weeks 1–20, 2019, with rates for 2018 and 2019 (2018 rate included for comparison)

Two weeks after cases of ILI peaked in Guam, the rate of ILI in CNMI began to increase, nearly doubling in 2 weeks (**Fig. 3**). The ILI rate increased consistently through week 16, reaching 9.3% (117/1254) of outpatient encounters. The number of confirmed cases rose from week 11 onwards, peaking during week 15 at 50 cases. Much like Guam, CNMI started the year with a higher number of confirmed cases caused by influenza A. In week 8, however, the number of confirmed cases caused by influenza B viruses for the first time exceeded the confirmed cases caused by influenza A. From week 7 to 20, 87% (293/338) of confirmed cases were caused by influenza B. Four specimens collected during weeks 16–17 were sent to the Guam Public Health Laboratory for serotyping, and all were identified as influenza B/Victoria (**Table 2**).

[**insert** Figure 3]

**Table 2:** Number of positive influenza specimens submitted for further testing, by lineage, US-affiliated Pacific Islands, weeks 1–20, 2019

Jurisdiction	Influenza type		Total no. of specimens tested
	A(H1N1)pdm09 and A(H3N2)	B/Victoria	
Commonwealth of the Northern Mariana Islands	0	4	4
Federated States of Micronesia (Federated States of)	0	2	2
Republic of the Marshall Islands	0	7	7

The age range among confirmed cases of influenza B/Victoria was 7 months to 11 years, consistent with the range in Guam. Those aged < 20 years accounted for 76% (770/1007) of cases of ILI from week 7 to 20. During that period, 46% (462/1007) of ILI cases occurred among people aged 5–19 years, with weekly percentages of ILI occurring in this age group ranging from 31% (19/62) to 57% (36/63). Only 5% (48/1007) of ILI cases occurred in those aged (*3*)50 years.

The population-wide vaccination rate in CNMI from August 2018 through week 20 of 2019 was 35% among those younger than 5 years (CNMI Commonwealth Health care Corporation, Division of Public Health Services Immunization Program, unpublished data, 2019). However, this is likely an overestimation, as it does not include those who received the second dose recommended for younger children. Among confirmed cases aged 0–4 years detected during weeks 8–18, 95% (84/88) were unvaccinated, although 14% (12/84) of these were too young for vaccination. Among cases aged 5–19 years, 86% (110/128) were unvaccinated.

### Federated States of Micronesia

Data from FSM indicate similar patterns to those in Guam and CNMI. The number of ILI encounters increased from week 11 to 15, when encounters peaked at 370, or approximately 2.7 times the year-to-date average of 136 ILI encounters per week. In week 14, there were 294 cases of ILI, approximately 1.8 times the 4-week average of 167 cases. For weeks 12–18, cases of ILI were above the year-to-date average. There were six confirmed cases: three of influenza A and three of influenza B. The influenza B viruses were all detected during weeks 14–15 in cases with an age range of 8–29 years. Of the two specimens from Yap subtyped by the Guam Public Health Laboratory, both were influenza B/Victoria (**Table 2**).

The increase in ILI cases in FSM appears to have been driven primarily by increased cases in Pohnpei, although this may have been amplified by missing data from other states. Pohnpei reported 67% (2068/3066) of FSM’s cases during weeks 1–20. Pohnpei’s ILI encounters nearly doubled from week 12 to 13, reaching 7.3% of outpatient encounters (153/2085). The ILI rate was above 10% for most of April and peaked at 14% (314/2239) in week 15. While ILI rates were not available for states other than Pohnpei, the number of ILI cases in Yap exceeded the threshold indicating heightened ILI activity during weeks 14–16.

### Republic of the Marshall Islands

**Figure 4:**
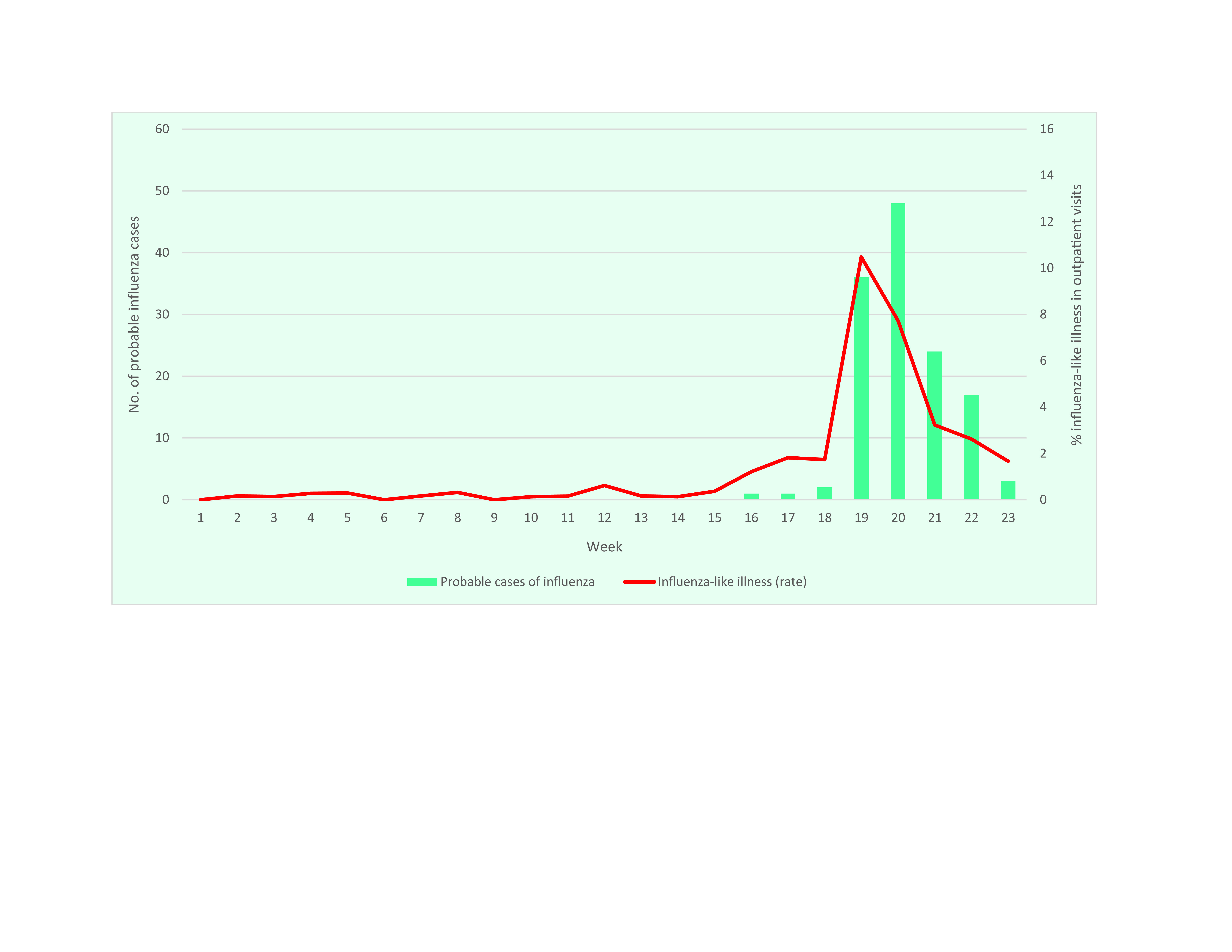
Number of reported cases of influenza-like illness and probable influenza, with rate of influenza-like illness, Majuro, Republic of the Marshall Islands, weeks 1–23, 2019

Influenza cases were reported in RMI later than in the other US-affiliated Pacific Islands and exceeded the expected ILI threshold only on the main island of Majuro. Only Majuro is connected to RMI’s health information system, which may impact the capacity to detect outbreaks. Within Majuro, the ILI rate remained < 1% until week 16 (**Fig. 4**). At the outbreak’s peak in week 19, the rate of ILI in outpatient encounters increased to 10.5% (111/1059), with 49% (54/111) of cases occurring among children aged < 5 years and 35% (39/111) among those aged 5–19 years. Only 3% (3/111) occurred among people aged (*3*)50 years. The rate of ILI detected in the outpatient department was 8% (40/497) in week 19 and 6.2% (29/467) the following week. Consistent with the age range affected by the outbreak, ILI rates were significantly higher in the Public Health/Maternal and Child Health Department, at 27.1% (69/255) in week 19 and 17% (38/224) in week 20.

There were 131 probable cases of influenza detected on Majuro during weeks 16–23. Among these, 61% (80/131) were among children aged < 5 years and 20% (26/131) were among those aged 5–19 years. Seven confirmed cases from week 19 were subtyped as influenza B/Victoria by the Hawaii State Laboratories Division (**Table 2**), with these confirmed cases ranging in age from 8 to 54 years.

[**insert** Figure 4]

Based on data extracted from the RMI national immunization information system, influenza vaccine coverage during the 2018–2019 season for Majuro was 66% for those aged < 20 years. Among the probable and confirmed cases, the overall vaccination rate was 5%, with a slightly higher rate (12%) among those aged 5–19 years.

## Discussion

Surveillance data identified unseasonal outbreaks of influenza during the spring of 2019 in four US-affiliated Pacific Islands: CNMI, FSM, Guam and RMI. Although historical data are limited, the number of cases of ILI reported at their peak in FSM in 2019 was three times higher than during the same week the previous year (104 cases in 2018 versus 322 in 2019), and the 4-week average at the height of the outbreak was 65% higher than during the same period in 2018 (103 in 2018 and 170 in 2019). Data from CNMI provide further evidence that the increase in ILI cases observed in 2019 was not consistent with recent regional trends, with the peak ILI encounter rate of 9.3% in spring 2019 for CNMI more than triple that during the same week in 2018 (2.7%). During the spring 2019 peak, the 4-week average for ILI encounters (106 encounters) was more than twice as high as during the same time in the previous year (43 encounters).

Of the 107 confirmed cases reported from Guam during 2019, 80% were influenza B/Victoria. Although only a few specimens from patients with ILI in CNMI, FSM and RMI were subtyped, all were found to be influenza B/Victoria. The timing and age distribution of these confirmed cases were also consistent with the confirmed cases from Guam. Previous studies have found higher rates of influenza B/Victoria than influenza B/Yamagata in younger age groups, with some highlighting that those of school age are at increased risk. (*3*-*5*) Contributing factors may include molecular differences and higher levels of genetic diversity in influenza B/Victoria viruses, which allow them to target younger people with less prior viral exposure. (*5*) Widespread circulation of both influenza B subtypes has been documented in the Pacific, with influenza B/Victoria predominant during 2010–2012 and 2016, and with influenza B/Yamagata predominant in 2013–2015 and 2017. (*6*) The outbreaks reported here perhaps indicate a resurgence of influenza B/Victoria over influenza B/Yamagata.

The four US-affiliated Pacific Islands in this report all lie in the tropical region between the equator and the 20th parallel north. Although the timing of these influenza outbreaks in these Micronesian islands was consistent with northern temperate climates, where influenza activity spikes in the winter months, (*6*) the emergence of influenza B/Victoria did not match the patterns of viruses circulating in the US mainland. During weeks 1–20, only 5% of influenza cases reported by the US to GISRS were caused by influenza B viruses. (*7*) The Oceania–Melanesia–Polynesia influenza transmission zone, of which all US-affiliated Pacific Islands are members, had similarly low levels of influenza B cases, according to the global reporting system. (*7*) Patterns of confirmed influenza cases in the broader WHO Western Pacific Region, driven in large part by data from China, were similar to those noted in this report: a decline in influenza A cases starting in January and influenza B increasing in early March, overtaking influenza A by the end of the month and remaining dominant through week 20. (*7*) Overall, 20% of cases reported to GISRS from WHO’s Western Pacific Region were influenza B, and 88% of these were influenza B/Victoria. (*7*)

The high volume of travellers to the US-affiliated Pacific Islands during the spring of 2019 could explain the distinct influenza peaks recorded. The rise of influenza B in Guam and CNMI that began around week 7 corresponded to high levels of visitors from Eastern Asian countries, (*8*, *9*) offering a plausible route of introduction. A total of 667 784 visitors arrived on Guam from January to May, mostly from the Republic of Korea (44%) and Japan (42%). (*9*) During that period, CNMI recorded 188 147 visitors, 47% from China and 42% from the Republic of Korea. (*9*)

China reported 87% of the influenza cases in GISRS from WHO’s Western Pacific Region and exhibited trends similar to those of the islands reported here. (*7*) In mid-February, influenza B cases began to increase in China, and comprised 82% of cases by week 20. (*7*) Influenza B began appearing around the same time in the Republic of Korea, increasing to account for more than 90% of confirmed influenza cases during weeks 18–20. (*7*) No data were available on subtyped influenza B viruses from the Republic of Korea, but 92% of specimens from China were identified as influenza B/Victoria and only 2% were influenza B/Yamagata. (*7*)

The increase in influenza B cases observed later in 2019 in FSM and RMI compared with CNMI and Guam may be partially attributable to the lack of direct flights from Eastern Asia. RMI received 2049 visitors during January–March 2019, with arrivals peaking in March. (*10*) Data from previous years suggest that most visitors to RMI come from other Pacific Islands and North America, (*11*) and FSM’s visitors are primarily from the US. (*12*) However, Guam serves as a primary air transport hub for both FSM and RMI, which may have provided an opportunity for the introduction of influenza B. This would help explain the delays in peak activity, with Guam’s burden highest in week 13, followed by that in FSM in week 15 and in RMI in week 20.

The epidemiological evidence provided on the vaccination status of influenza cases has implications for immunization policy. The high attack rate among those aged < 20 years underscores the vulnerability of the young to seasonal influenza and reinforces the need for concentrated efforts to vaccinate this population. All four US-affiliated Pacific Islands in this report used influenza vaccines approved for the northern hemisphere that included a B/Victoria/2/87 virus (B/Colorado/06/2017-like) in both the trivalent and quadrivalent formulations. (*13*) Further testing would be needed to determine whether any differences between the vaccine formulation and the circulating strain could partially explain the higher attack rate among those aged < 20 years. Low vaccination rates in this age group are likely a contributing factor. The availability of data about the vaccination status of cases in CNMI and RMI provides support for the effectiveness of the vaccine, with 12–14% of cases aged 5–19 years having received the vaccine in those jurisdictions.

There were some limitations to our analysis. The data presented in this report were drawn from surveillance systems in four different US-affiliated Pacific Islands, each with unique data collection procedures. Although the Pacific Syndromic Surveillance System provides a standard definition of ILI, the way in which that definition is operationalized locally may result in disparate levels of data integrity. While surveillance based on WHO definitions distinguishes ILI from severe acute respiratory infection based on whether the case was hospitalized, (*14*) it is possible that some cases of severe acute respiratory infection were reported as ILI.

Because the jurisdictions represented in this report are characterized by their relatively remote locations, in some cases, limited public health infrastructure and completeness of reporting may have influenced the findings. It is possible that resource availability and physical distance from larger hubs reduced reporting from outer islands.

Those presenting with ILI were not systematically selected for rapid influenza testing, but were selected at the provider’s discretion. Heightened awareness of influenza activity may have influenced providers’ decisions to test and report patients presenting with ILI. However, consistent patterns of age distribution and influenza type in CNMI, where a large portion of ILI encounters were followed up with nasopharyngeal swabs, provide evidence that overall trends may not have been significantly impacted by nonrandom selection.

Classifying influenza cases into either influenza A or B was made possible through rapid testing, but only a small fraction of specimens was subtyped, and most of the subtyped specimens were from Guam. Beyond its lineage as an influenza B/Victoria virus, isolation of the exact strain circulating was not possible, which precludes determination of whether the virus was contained in the 2018–2019 influenza vaccine as well as precluding confirmation that the viruses were similar to those circulating in China, Japan or the Republic of Korea. Analyses based on the immunization status of cases were limited because this information was not routinely reported for ILI encounters in all jurisdictions.

## Conclusions

The ability of the sentinel surveillance system to detect influenza outbreaks in four US-affiliated Pacific Islands is a testament to the value of year-round surveillance for ILI because it ensures that clinical teams are informed about circulating respiratory infections. Epidemiological analysis identified the age groups most at risk, aiding both clinical and public health responses. Although influenza B viruses are not considered to have pandemic potential, identifying circulating strains is important, as demonstrated by the increased burden seen in younger age groups. Understanding changing influenza patterns helps in evaluating immunization effectiveness and gaps in coverage to protect the population from an undue burden of disease.
